# Rare case of Torsades de Pointes in severe hypothyroidism: literature review and challenges in management

**DOI:** 10.1186/s12245-022-00417-5

**Published:** 2022-03-14

**Authors:** Berlin Lee, Wei Feng Lee, Beng Leong Lim

**Affiliations:** Emergency Department, Ng Teng Fong General Hospital, 1 Jurong East Street 21, Singapore, 609606 Singapore

**Keywords:** Hypothyroidism, Arrhythmia, Torsades de Pointes, Long QT interval

## Abstract

**Background:**

Hypothyroidism can manifest as several important cardiac abnormalities. There are few reports of ventricular dysrhythmias (VDs) in hypothyroidism. We described a rare case of VDs in severe hypothyroidism and reviewed the literature behind its management.

**Case presentation:**

A 67-year-old gentleman, with poor compliance to treatment for Hashimoto’s thyroiditis, presented with palpitations to the Emergency Department. He had runs of non-sustained ventricular tachycardia (NSVT). He was treated with intravenous (IV) amiodarone and admitted to the intensive care unit for observation. He then developed recurrent Torsades de Pointes (Tdp) despite treatment with several anti-arhythmics. He required electrical cardioversion and eventual transvenous overdrive pacing (OP). VT recurred while he was on OP. VT resolved and he was weaned off OP only after adequate thyroid hormone replacement.

**Conclusions:**

VDs, including NSVT, Tdp, and VT, are rare and potentially lethal in hypothyroidism. Our case demonstrates important challenges in the management of severe hypothyroidism. Here, VDs are often refractory to treatment with drugs and electrical means. The choice(s) of anti-arrhthymics requires careful consideration and can be difficult before thyroid function tests are known. Amiodarone use should be cautioned as it is associated with thyroid dysfunction and QT interval prolongation.

There is no literature to guide thyroid hormone replacement in this disease. Aggressive replacement is associated with adverse cardiovascular effects. Our case showed a fine balance between the risk of rapid thyroid hormone replacement and the urgency to terminate VDs. Its administration should be carefully monitored amidst bridging strategies like electrical cardioversion and OP to manage life-threatening VDs.

## Background

Hypothyroidism can manifest as several important cardiac abnormalities. There are few reports of ventricular dysrhythmias (VDs) in hypothyroidism. We described a rare case of VDs in severe hypothyroidism and highlighted important challenges in decision-making, treatment of life-threatening VDs, and concurrent thyroid hormone replacement. These processes are often complex and have limited prior literature.

## Case report

**Fig. 1:**
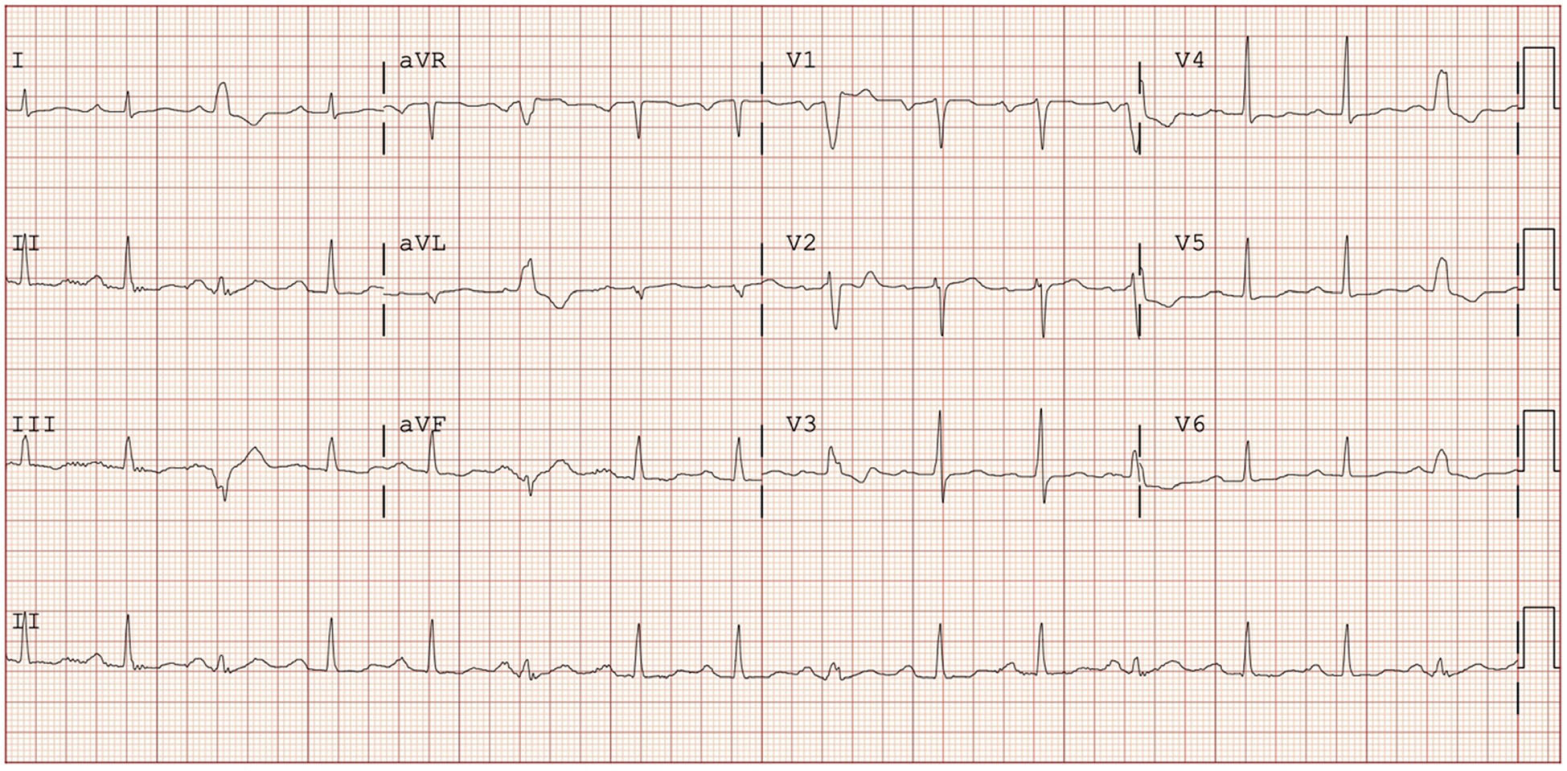
Electrocardiogram showing sinus rhythm with premature ventricular contractions

A 67-year-old Chinese gentleman, with Hashimoto’s thyroiditis and poorly compliant to treatment, presented with palpitations to the Emergency Department (ED). His vital signs were afebrile, blood pressure 154/106 mmHg, and heart rate 102 beats/minute. Physical examination was unremarkable. Initial electrocardiogram showed sinus rhythm with premature ventricular contractions (Fig. 1).

**Fig. 2:**
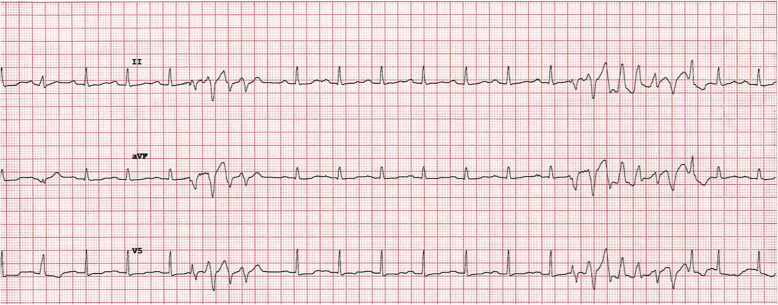
Electrocardiogram showing recurrent non-sustained ventricular tachycardia

He developed recurrent runs of non-sustained ventricular tachycardia (NSVT) (Fig. 2). An intravenous (IV) loading dose of 150 mg of amiodarone followed by an infusion of 300 mg over 8 h was administered. Initial investigations showed severe primary hypothyroidism with free thyroxine (FT4) < 5 picomol/liter (*p*mol/L) and thyroid-stimulating hormone (TSH) >100 milliunits/liter (mU/L). Serum potassium and magnesium levels were normal.

**Fig. 3:**
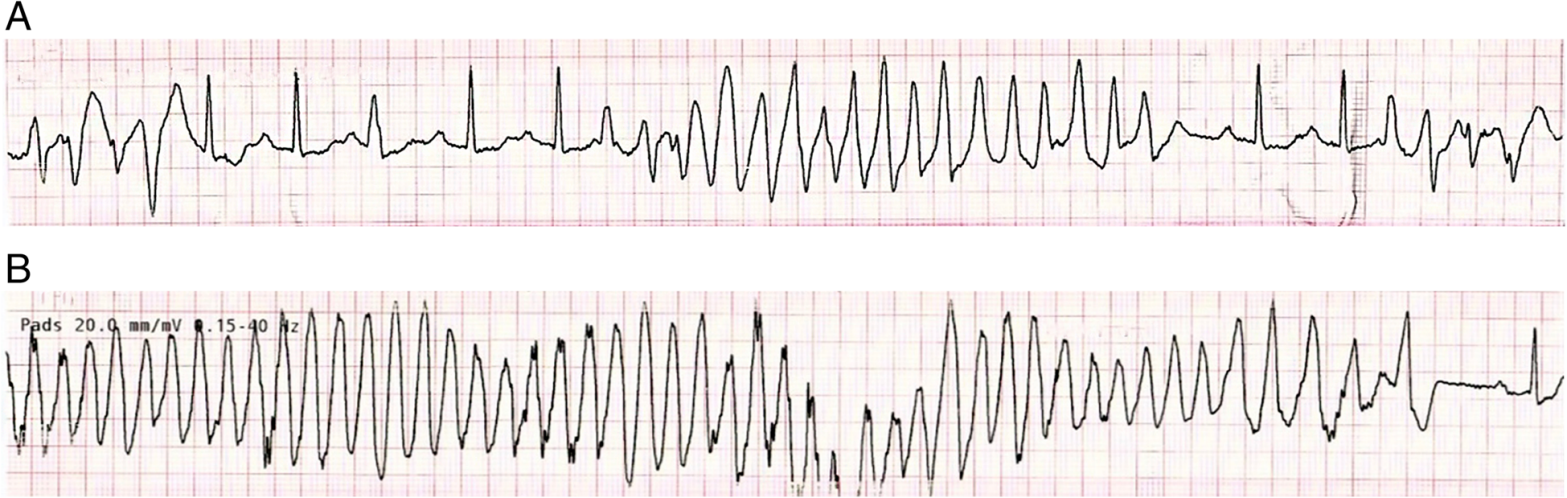
**A** Polymorphic ventricular tachycardia with preceding prolonged QT interval. **B** Prolonged run of polymorphic ventricular tachycardia

He was admitted to the intensive care unit (ICU) where he had multiple short runs of polymorphic ventricular tachycardia (VT) with prolonged QT interval (Fig. 3A, B). He was given IV magnesium sulphate, IV lignocaine, and oral bisoprolol. A diagnosis of Torsades de Pointes (Tdp) secondary to hypothyroidism was made. IV levothyroxine replacement was commenced with 300 mg initially followed by 100 mg daily for 3 days.

**Fig. 4:**
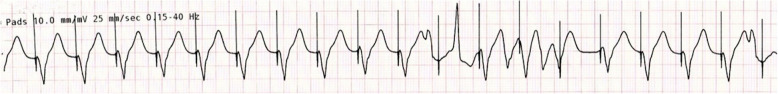
Electrocardiogram showing transvenous overdrive pacing

**Fig. 5:**
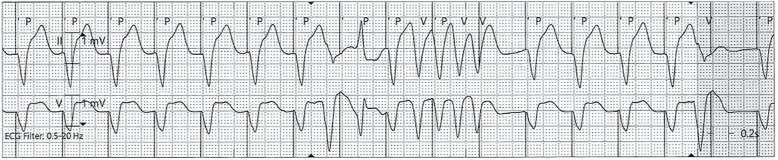
Electrocardiogram showing ventricular tachycardia escape despite overdrive pacing

He developed further episodes of Tdp with hemodynamic compromise and required repeated electrical cardioversion. He was intubated and transvenous overdrive pacing (OP) was performed on day 2 (Fig. 4). VT recurred despite OP (Fig. 5). Further investigations showed total triiodothyronine (T3) <0.31 nanomoles/liter and free T3 <2.3 pmol/L. Liothyronine of up to 20 mcg per day was added to levothyroxine 150 mg orally on day 4. He was subsequently extubated. VT did not recur when his thyroid hormones were sufficiently replaced on day 7 (FT4 8.3 pmol/L, TSH 53.22 mU/L, FT3 2.6 pmol/L). OP was ceased on day 8. He was discharged from ICU and hospital on days 12 and 16, respectively.

## Discussion and conclusion

Hypothyroidism is associated with electrocardiographic changes like sinus bradycardia, QT interval prolongation, and heart blocks. In severe cases, VDs like NSVT and Tdp occur [1]. The incidence rates of VDs and VT in hypothyroidism are 6.58% and 2.63%, respectively [2]. There are relatively few case reports of malignant VDs in hypothyroidism [3].

Our case demonstrated important challenges when managing VDs in severe hypothyroidism. Here, VDs are refractory to treatment with anti-arrhythmics, electrical cardioversion, and OP until thyroid hormones are sufficiently replaced. The choice(s) of anti-arrthymics requires careful consideration. This decision can be more difficult before thyroid function test (TFT) results are available and when emergency physicians (EP) are unfamiliar with the spectrum of VDs in hypothyroidism.

Caution is needed when using amiodarone for treating VDs in hypothyroidism. Amiodarone can result in unpredictable thyroid dysfunction through the Wolff-Chaikoff effect (reduced hormone synthesis) or its innate iodine load (enhanced hormone synthesis) [4]. Amiodarone can also prolong QT interval and promote arrhythmias like Tdp [5]. EP need to maintain a high level of suspicion for severe hypothyroidism with history of poor compliance to thyroid hormone replacement before TFT results are known. Safer anti-arrhythmics like lignocaine (which can shorten QT interval) can be considered [5, 6].

Electrical means like cardioversion and OP are used in our case to manage refractory VDs (Tdp and VT) before hypothyroidism is corrected. Medical chronotropic therapy like isoproterenol infusion can also be considered as increasing heart rate decreases QT interval [7]. These measures are bridging strategies to treat life-threatening VDs before adequate thyroid hormone replacement.

Finally, our case demonstrated a fine balance between the risk of aggressive thyroid hormone replacement and the urgency to terminate VDs especially Tdp and VT. There is no current consensus on the recommendations for thyroid hormone replacement in hypothyroid patients with VDs. The replacement regime is extrapolated from that recommended in myxoedema coma, another manifestation of severe hypothyroidism.

Replacement of thyroid hormone can be started with IV thyroxine at a loading dose of 300 to 500 mg followed by 1.6 mg/kg/day in myxoedema coma [8]. Triiodothyronine replacement is recommended after thyroxine replacement fails and can be started at a dose of 10 to 25 mg followed by 2.5 to 10 mg every 8 h [8]. However, there is higher mortality risk when thyroxine doses exceed 500 mg or triiodothyronine doses exceed 75 mg daily [8]. Excessive thyroid hormones are associated with adverse cardiovascular effects such as increased risks of supraventricular arrhythmia like atrial fibrillation and may precipitate heart failure, embolic stroke, or death [9]. However, Tdp, if left unchecked, can also deteriorate into ventricular fibrillation and lead to death [10]. Hence, thyroxine replacement should be carefully monitored and administered in patients with hypothyroidism and VDs. Triiodothyronine administration can be considered early if VDs are refractory to both pharmacological and electrical means. This often requires multi-disciplinary management involving the EP, intensivist, cardiologist, and endocrinologist.

## Data Availability

Not applicable.

## Abbreviations

ED: Emergency Department
EP: Emergency physicians
FT4: Free thyroxine
ICU: Intensive care unit
IV: Intravenous
NSVT: Non-sustained ventricular tachycardia
OP: Overdrive pacing
T3: Triiodothyronine
Tdp: Torsades de Pointes
TFT: Thyroid function test
TSH: Thyroid-stimulating hormone
VD: Ventricular dysrhythmia
VT: Ventricular tachycardia
